# Coagulation in Brain Tumors: Biological Basis and Clinical Implications

**DOI:** 10.3389/fneur.2019.00181

**Published:** 2019-03-18

**Authors:** Chiara Mandoj, Luigi Tomao, Laura Conti

**Affiliations:** ^1^Clinical Pathology, IRCCS Regina Elena National Cancer Institute, Rome, Italy; ^2^Department of Hematology/Oncology, IRCCS Bambino Gesù Children Hospital, Rome, Italy

**Keywords:** thromboembolism, glioblastoma, tissue factor, oncogenes, TF microparticles

## Abstract

Cancer patients commonly show abnormal laboratory coagulation tests, indicating a subclinical hypercoagulable condition that contribute to morbidity and mortality. The hypercoagulation status not only increases the risk of thromboembolic events but also influences the tumor biology promoting its growth and progression by stimulating intracellular signaling pathways. Recent molecular studies characterized the role of oncogene and suppressor gene in activating clotting pathways, as an integral feature of the neoplastic transformation. It is now clear how haemostatic processes, activated by cancer cells harboring oncogenic mutations, rely on the molecular profile of a particular malignancy, an aspect particularly evident in the differential coagulome profiles showed by different molecular subtypes of brain tumors, such as glioblastoma and medulloblastoma. This review focuses on the biological and clinical aspects of haemostasis in cancer with particular regard on brain tumors.

## Introduction

Thromboembolic events are well-recognized complication of malignant disease and are associated with significant mortality and reduced survival. Very often, at the same extent, an “idiopathic” venous thrombosis can exacerbate a latent cancer not diagnosed until then. The first evidence of an higher risk to develop a venous thromboembolism (VTE) in cancer patients was documented initially in 1865 by Armand Trousseau, who first reported the risk of migratory thrombophlebitis in occult malignancy (Trousseau's syndrome) (1). In 1878, instead, Theodor Billroth confirmed the role of VTE in the metastatic process demonstrating the presence of tumor cells in patient-derived thrombi (2).

VTE occurs in the 4–20% of cancer patients, but its frequency reach the 50% when also postmortem examinations are considered; however, approximately the 20% of all cases of VTE derives from cancer patients.

The clinical manifestations of haemostasis activation vary from a subclinical asymptomatic hypercoagulable state to manifest thrombosis of the large vessels. The pathogenesis of cancer-associated coagulopathy is complex and involves various mechanisms, however all of them are linked to abnormalities of Virchow's triad: stasis of the blood flow (prolonged hospitalization, mechanical tumor-derived compression of blood vessels); endothelial injury (tumor invasion, central venous catheters insertion, administration of chemotherapic agents, radiotherapy, and growth factors); hypercoagulability (release of tumor-associated procoagulants molecules and cytokines) (3–6).

## Pathogenesis of Thrombosis in Cancer

The mechanisms bearing the activation of the haemostatic system in cancer are complex and likely multifactorial, involving some important biological and clinical factors, such as the primary cancer site and the stage or extent of the disease, but also the anticancer treatments, including chemotherapy, hormone therapy, anti-angiogenic therapy, and combination regimens; finally the practice of central venous catheter indwelling and the surgery (7–12).

Thrombotic events occur more frequently in patients with pancreas, lung and colon cancer, as well as in haematologic malignancies and, when adjusted for disease prevalence, in ovary and brain tumors (13–16).

## Prothrombotic Mechanisms

Malignant cells display prothrombotic properties and promote the development of a persistent hypercoagulability status by expressing procoagulant and fibrinolytic molecules. Cancer cells also release proinflammatory and proangiogenic cytokines, such as interleukin 1β (IL-1β), tumor necrosis facror α (TNF-α) and vascular endothelial growth factor (VEGF), basic fibroblast growth factor (bFGF), respectively. Finally, they surely interact with endothelial cells, leucocytes and platelets through the expression of several adhesion molecules (17–19).

Tumor cells directly activate haemostasis by producing tissue factor (TF), the principal activator of the blood coagulation process, promoting thrombin generation, and facilitating fibrin deposition creating a microenvironment, in solid tumors, that protects cancer cells from the immune system attack (15). Circulating TF can actually be detected in cancer patients plasma samples associated to microparticles (MPs). These are submicrometric vesicles, derived from activated or apoptotic cells, carrying both TF and phosphatidylserine on their surface, that promotes the coagulation process (20–22). Elevated levels of TF-MPs are associated with higher risk of VTE in cancer patients and correlate with D-dimer levels and other biomarkers of coagulation activation (23–27). In addition, a high rate of TF-MPs activity in patients-derived plasma correlates with the diagnostic and prognostic features of pancreatic cancer, revealing the less differentiated and more aggressive forms (28). This suggests that TF-MPs not only may contribute to development of cancer-associated thrombosis but also to tumor progression (27).

**Figure 1:**
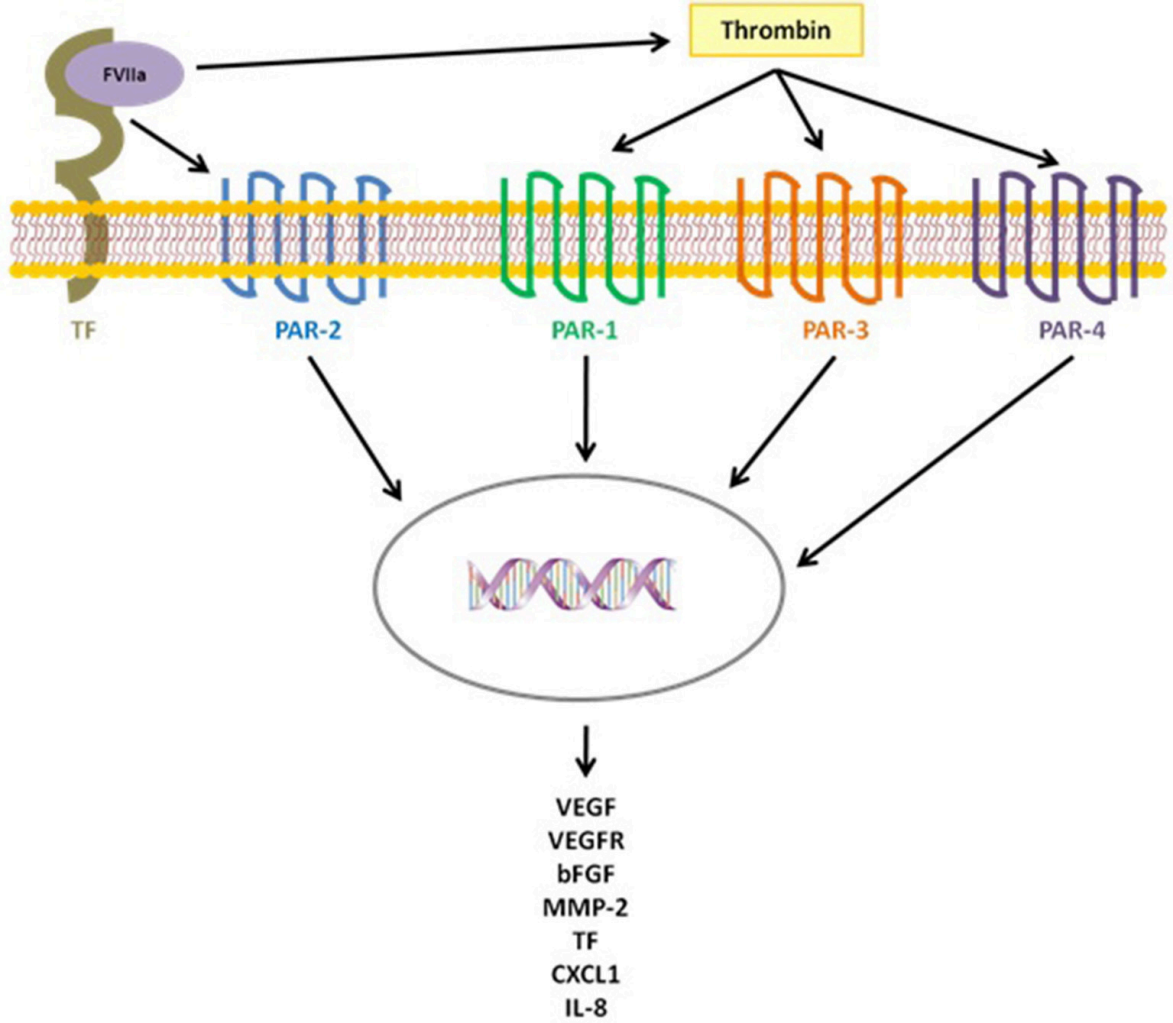
TF triggers several pathogenic events, crucial for cancer progression, both directly, activating PAR-2 after binding FVIIa, and indirectly activating the coagulation cascade with thrombin generation and the following activation of PAR-1,3,4.

TF release, and the following thrombin activation, not only trigger the hemostatic process but also several pathogenic events crucial for cancer progression, such as cell survival and invasion, as well as angiogenesis processes, through the activation of the protease-activated receptor (PARs) proteins, a group of membrane receptor proteins (PAR 1–4), expressed by tumor cells and vascular cells, that mediate cell activation via G protein pathways (29–32). The cytoplasmic domain of TF, in fact, participate in propagating the intracellular signaling initiated by PARs activation, affecting cancer cell behavior. In particular, the TF/factor VII (FVII) complex proteolytically activates PAR-2 and the formed TF-FVII-PAR-2 complex guides the expression of tumor metastasis by modulating genes that regulate cell survival and migration, inducing, at the same time, the release of pro-angiogenic factors, such as CXCL1, VEGF, and IL-8. Likewise, the interaction of thrombin with PAR-1, PAR-3, and PAR-4 triggers several signaling pathways in cancer cells ending with the overexpression of many angiogenesis-related genes, like bFGF, TF, VEGF, VEGF receptor (VEGFR), and metalloproteinase 2(MMP-2) (Figure 1) (33, 34). Another way of angiogenesis activation resides in the ability of cancer cells, as well as other cells of the tumor microenvironment (e.g., the pericytes), to express the NG2 proteoglycan, which promotes β-1 integrin signaling activation and the downstream FAK and PI3K/Akt pathways. Several studies, in fact, demonstrated that NG2 expression by tumor and stroma cells contributes to tumor progression and correlates with malignancy, triggering cancer aggressiveness and the generation of new tumor-associated blood vessels (35–37).

Tumor cells, at the same time, can synthesize all of the components of the fibrinolytic system, such as urokinase-type plasminogen activator (uPA) and its receptor (uPAR), tissue-type plasminogen activator (tPA) and plasminogen activator inhibitors 1 (PAI-1) and 2 (PAI-2). Several studies demonstrated that these proteins may facilitate tumor invasion and metastasis, laying the foundation for their use as potential biomarkers, being detectable both in plasma samples and in histological sections. It is actually well known, *in vivo*, that high levels of uPA promote matrix degradation and cancer progression and that high levels of PAI-1 expression, in particular in breast cancer, is associated with poor prognosis. At the same time a poor prognosis was also found in patients with breast, ovarian and colorectal cancer that showed high pre-operative plasma levels of uPAR and PAI-1 (38–41).

Tumor cells can also drive platelet activation (inducing an overexpression of CD62P) and aggregation, directly by cell-cell adhesion, indirectly by releasing activation factors (such as ADP and protease) and by inducing thrombin generation via coagulation activation. Circulating platelets contain several angiogenesis-regulating agents (such as VEGF, PDGF, bFGF, EGF, HGF, IGF1, TGFβ) that are released upon their activation. The interaction between tumor cells and platelets, as well as leukocytes and endothelial cells, during the metastatic progression occur thanks to the expression of different integrins (i.e., E, P, and L) that facilitate cancer cell adhesion, diapedesis, and the formation of the metastatic lesions (42–44).

Both tumor cells, through the activation of prothrombotic pathways, and the host cell, through their ability to trigger the inflammatory response, take part in the pathogenesis of cancer-related thrombosis, revealing the tight interrelation between inflammation and haemostasis, two overlapping processes that notably affect each other (45, 46).

## Oncogenic Pathways and the Coagulant Phenotype in Cancer Cells

The role of cancer-derived hemostatic system deregulation in tumor progression and metastasization is well-characterized. Cancer cells, with their altered gene profile, promotes a selective advantage for their own survival in the tumor niche, as, thanks to a procoagulant activity, they are able to induce the formation of fibrin scaffolds in which the potential to anchor and invade is promoted. At the same time, coagulation proteins recognized from cancer cell receptors can induce the propagation of intracellular signals favoring tumor growth and progression. Until now coagulopathy thrombosis and Trousseau syndrome have been considered as “unspecific” consequences of cancer related disruption of tissue anatomy and vascular continuity, representing the very first element of tissue response to injury.

Recent molecular studies demonstrated that neoplastic transformation, induced by oncogene and suppressor gene mutations, may activate clotting pathways, both *in vitro* and *in vivo* (47–51). For example it has been reported, in colorectal cancer, that mutations of K-ras and p53 (associated to p53 loss of function) are associated with a high TF expression. Moreover, an association between circulating MP-TF activity levels and the mutational status of cancer cells was found (47, 52). In the same way a TF upregulation was found in squamous cell carcinoma (SCC) and glioblastoma multiforme (GBM), especially when mutations of the epidermal growth factor receptor (EGFR) and loss of E-cadherin occur (48, 53). It was then demonstrated that in cancer cells a higher EGFR expression, together with the overexpression of the EGFR variant III (EGFRvIII), trigger the TF expression. On the other side, when phosphatase and tensin homolog (PTEN) is restored in these cells, causing the inhibition of the phosphatidylinositol 3-phosphate kinase (PI3PK) and mitogen-activated protein kinase (MAPK) pathways, a downregulation of the EGFR-dependent TF expression was found (48, 54). In a mouse model of sporadic tumorigenesis, instead, the activation of the oncogene MET brought to the generation of spontaneous multifocal hepatocellular carcinoma (HCC), together with a lethal thrombohemorrhagic syndrome as a consequence of cyclooxygenase-2 (COX-2) and PAI-1 up-regulation, since some clinical symptoms got milder when treatment with their inhibitors was performed (49, 55).

**Figure 2:**
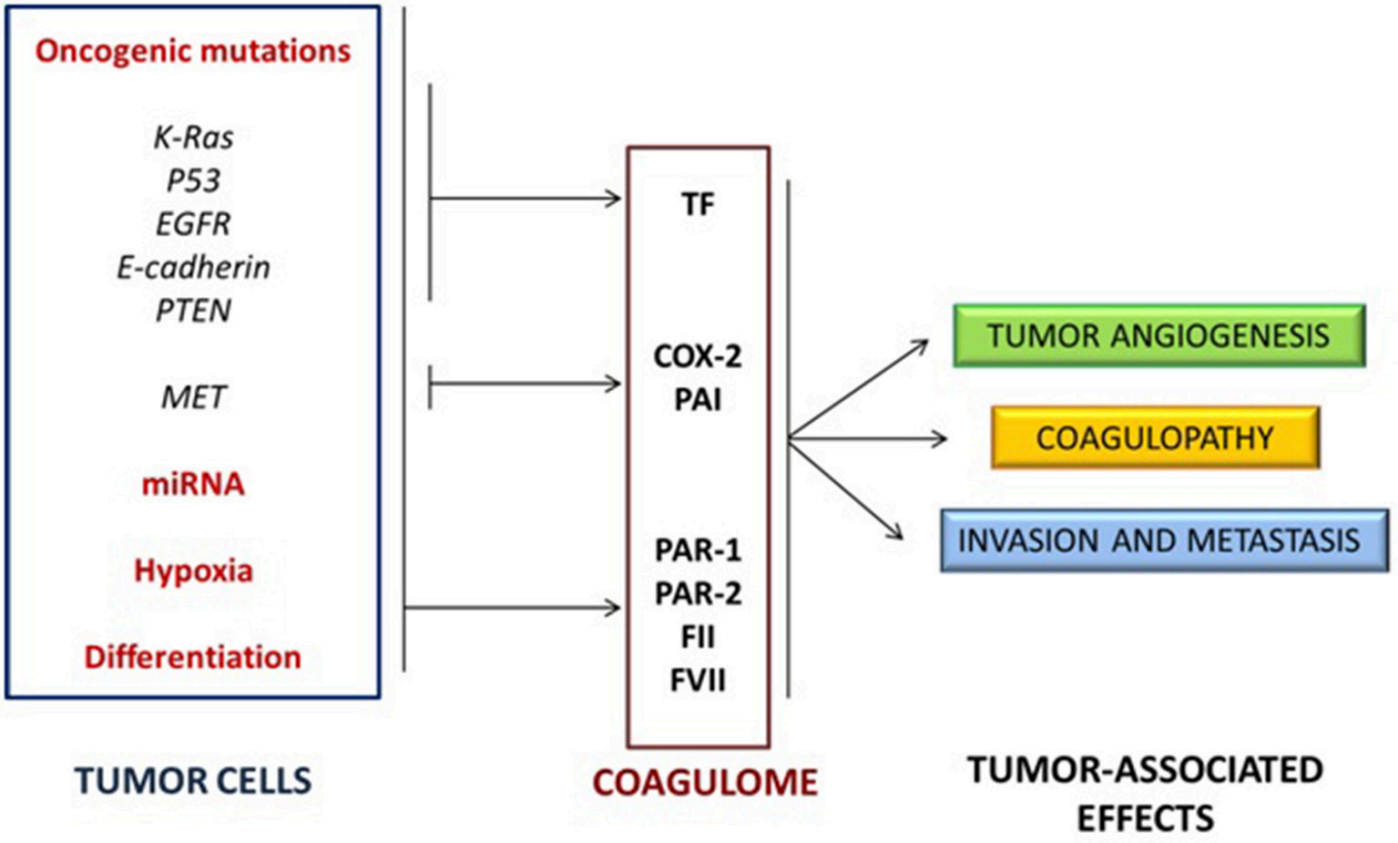
Activation of oncogenes and inactivation of tumor suppressor genes cooperate with non-coding RNA expression, hypoxia and cellular differentiation to control the expression of several proteins of the coagulation/fibrinolytic system and platelet activation.

These findings suggested that specific cancer cell phenotypes may affect the coagulation system, that the deregulation of haemostasis in tumors microenvironment is not “unspecific” and that the activation of oncogenes (such as EGFR, MET, or RAS) and the inactivation of tumor suppressor genes (such as PTEN or p53) directly affect the expression of hemostasis-controlling genes (50, 51). Other studies pointed out how oncogenic mutations and non-coding RNAs (e.g., microRNAs) can cooperate with hypoxia and cellular differentiation to control the expression of several proteins of the coagulation system, such as TF, PAR-1 and PAR-2, FII and FVII, as well as molecules of the fibrinolytic system and platelet activation (56) (Figure 2).

One of the most fascinating theory in this field focuses on the differential coagulome profiles expressed by different tumor subtypes, such as medulloblastoma (MB), GBM, and other tumors, trying to reveal the possible linkage between tumorigenesis and specific procoagulant phenotypes expressed by cancer cells (57). GBM is the most lethal type of primary brain tumor and is associated with florid angiogenesis, thrombotic complications and up-regulation of TF. In the last few years emerged that different molecular subtypes of GBM (i.e., proneural, neural, classical, and mesenchymal) also showed specific coagulomes features. In the classical GBM, for example, cancer cells overexpress the TF, showing an important procoagulant phenotype, hypothetically driven by the expression of the oncogenic EGFR and its mutant form EGFRvIII. Classical GBM cells, actually, do not show only TF overexpression, but also higher levels of PAR-1 and PAR-2, as well as an ectopic synthesis of FVII. A particular study, on the other side, elegantly demonstrated that the overexpression of TF and the procoagulant activity of GBM cells, after the inactivation of PTEN, are triggered only under hypoxic conditions or together with the EGFRvIII expression, demonstrating how the activation of specific oncogenic pathways, rather than individual mutations, may drive tumor cells to express a particular procoagulant phenotype (48, 53, 54).

The proneural subtype of GBM, a tumor often bearing isocitrate dehydrogenase 1 (IDH1) mutations, is instead associated with lower TF expression. IDH1-mutated cancer cells produce high levels of D-2-hydroxyglutarate (D-2-HG), a molecule that rapidly inhibit platelet aggregation and the related blood clotting events, in a calcium-dependent way (58). It is interesting to notice, in this case, that patients with mutated IDH1 do not suffer from thrombotic events, while the 26–30% of wild-type patients develop a VTE, indicating that the IDH1 mutated form give an antithrombotic potential to GBM cells (59). In addition to this, mutant IDH1 is associated with lower PI3K signaling and podoplanin (PDPN) expression (60). PDPN is a sialomucin-like glycoprotein that binds C-type lectin receptor type-2 (CLEC-2) and causes platelet aggregation. A recent study showed that PDPN expression in GBM is associated with risk of VTE and mortality (61).

Finally, in the mesenchymal form of GBM, in which a pronounced inflammatory status was found, TF is downregulated, while high levels of PAI-1, uPA, uPAR, EPCR, and Thrombomodulin were observed. All these data indicate that GBM cancer cells, characterized by a particular mutational status, may induce the activation of the coagulation system, influencing angiogenic processes and impacting tumor progression (62–64).

Similar studies have been conducted on other types of brain tumors. Pediatric brain malignancies, for example, are not procoagulant tumors, with the MB representing the most common and aggressive one. Molecular classification of MB distinguishes four disease subgroups, including those showing abnormalities in the wingless pathway (WNT) or in the sonic hedgehog pathway (SHH), along with the group 3 (G3) and 4 (G4). Among them, G3 accounts for patients with the poorest prognosis, especially when the amplification of the proto-oncogene MYC is found. Analyzing the coagulation status in MB, a TF overexpression was found only in the SHH subgroup, in association with a higher expression of MET and with altered responsiveness to thrombin and changes in angiogenic and inflammatory factors (63, 65, 66). SSH tumors are highly vascular but do not show an association with systemic thrombosis, confirming that the coagulant phenothype can contribute and influence the tumor biology in different ways.

The sequence of oncogenic mutations related to the different stages of the disease (i.e., pre-malignant, indolent and aggressive) seems to be also correlated with the vascular abnormalities that induce the neoangiogenesis process (62, 64). Oncogenic pathways, in fact, influence the recruitment of inflammatory cells which may exhibit proangiogenic and procoagulant phenotypes, inducing the expression of TF not only in malignant cells but also ectopically in tissue that normally do not produce coagulation factors. These factors modulate the biogenesis of procoagulant extracellular vesicles (EVs) and TF-MPs that, entering the general circulation, promote the metastatic spread (25, 67, 68).

In 1984, instead, a particular study focused on the association between head injuries and the incidence of GBM, concluding that some traumas may increase the risk to develop brain tumors (69). Karpatkin was the first that postulated that thrombin may trigger growth in dormant cancer cells awaken by tissue injury (e.g., head trauma) or the following repair processes (i.e., clotting and inflammation) (70). Certain human GBM cell lines, in fact, exhibit a permanent dormant phenotype, when injected in mice, expressing low TF levels and procoagulant activity. When a concomitant expression of EGFRvIII occur, however, a higher expression of TF is found, together with a highly procoagulant and more aggressive phenotype. Interestingly, inducing high TF levels by transfection, in absence of EGFRvIII, also interrupted the dormant state but in a delayed and less aggressive manner. These observations suggested that TF could play a role in regulating cellular properties inducing changes in the tumor microenvironment, linked to an epigenetic evolution of tumor cells that switch from a “dormant” to a malignant phenotype, not only expressing a procoagulant behavior (51).

## Conclusions

Malignant disease is characterized by a hypercoagulable state and several data have improved our knowledge about the relationship between tumor and thromboembolic risk, intimately linked to the processes of tumor growth, progression and metastasis. Haemostatic mechanisms are activated by cancer cells harboring oncogenic mutations in a way that seems to be dependent from the tumor molecular subtype. Markers of coagulation activation, such as cross-linked fibrin degradation products (D-dimer), prothrombin fragment 1+2 (PF1+2), Thombin-antithrombin complex (TAT), von Willebrand factor (vWF), circulating procoagulant MPs, and the manifestation of thrombosic events are associated with tumor progression and prognosis (71–73). Genetic strategies targeting TF, thrombin, FVII and PARs have been successfully used as modulators of tumor pathogenesis and progression, suggesting that deregulation of the hemostatic system may influence tumor microenvironment in several ways. In this scenario it would be of great importance to develop risk assessment models to predict thrombosis events in cancer, in order to identify high-risk patients and lead to the definition of target-specific treatment for both cancer and thrombosis.

## Author Contributions

LC and CM elaboration of the review. LT analysis of the literature and revision of manuscript.

### Conflict of Interest Statement

The authors declare that the research was conducted in the absence of any commercial or financial relationships that could be construed as a potential conflict of interest.
